# Entomology beyond research and education: 2022 student debates

**DOI:** 10.1093/jisesa/iead036

**Published:** 2023-06-06

**Authors:** Ramandeep Kaur Sandhi, Victoria Pickens, Elizabeth Bello, Sarah Elzay, Sara Salgado, Kayleigh C Hauri, John J Ternest, Natalie Constancio, Scott Gula, Olivia M Gearner, Magdeline Anderson, Molly Edeburn, Brandon Hall, Jacqueline Maille, Mollie Toth, Arjun Khadka, Ethan Doherty, Tyler Musgrove, Tiago Silva, Alexia Desoto, Emily Rampone, Dowen Jocson, Mario Luppino, Kellen Pautzke, Camille Wagstaff

**Affiliations:** FMC Corporation, Stine Research Center, 1090 Elkton Rd., Newark, DE 19711, USA; Department of Entomology, Kansas State University, Manhattan, KS 66506, USA; Department of Entomology, University of Illinois at Urbana-Champaign, Urbana, IL 61801, USA; Department of Biological Sciences, Fort Hays State University, Albertson Hall 302, Hays, KS 67601, USA; Department of Entomology and Nematology, University of Florida, Fort Pierce, FL 34945, USA; Department of Entomology, Michigan State University, East Lansing, MI 48824, USA; Department of Entomology and Nematology, University of Florida, Gainesville, FL 32608, USA; Department of Entomology, Michigan State University, East Lansing, MI 48824, USA; Department of Entomology, Purdue University, West Lafayette, IN 47907, USA; Department of Entomology, Purdue University, West Lafayette, IN 47907, USA; Department of Entomology, Kansas State University, Manhattan, KS 66506, USA; Department of Entomology, Kansas State University, Manhattan, KS 66506, USA; Department of Entomology, Kansas State University, Manhattan, KS 66506, USA; Department of Entomology, Kansas State University, Manhattan, KS 66506, USA; Department of Entomology, Kansas State University, Manhattan, KS 66506, USA; Department of Entomology, Louisiana State University, 404 Life Sciences Building 70802, Baton Rouge, LA 70803, USA; Department of Entomology, Louisiana State University, 404 Life Sciences Building 70802, Baton Rouge, LA 70803, USA; Department of Entomology, Louisiana State University, 404 Life Sciences Building 70802, Baton Rouge, LA 70803, USA; Department of Entomology, Louisiana State University, 404 Life Sciences Building 70802, Baton Rouge, LA 70803, USA; Department of Entomology, Louisiana State University, 404 Life Sciences Building 70802, Baton Rouge, LA 70803, USA; Department of Entomology, Washington State University, PO Box 646382, Pullman, WA 99164, USA; Department of Entomology, Washington State University, PO Box 646382, Pullman, WA 99164, USA; Department of Entomology, Washington State University, PO Box 646382, Pullman, WA 99164, USA; Department of Entomology, Washington State University, PO Box 646382, Pullman, WA 99164, USA; Department of Entomology, Washington State University, PO Box 646382, Pullman, WA 99164, USA

**Keywords:** forensic entomology, art, culture, research, ethics

## Abstract

The 2022 student debates of the Entomological Society of America (ESA) happened during the Joint Annual Meeting of the Entomological Societies of America, Canada, and British Columbia in Vancouver, BC, and addressed entomological aspects beyond research and education. The Student Debates Subcommittee of the ESA Student Affairs Committee and the participating student team members communicated for 8 months and prepared for the debates. The theme of the ESA meeting in 2022 was “Entomology as inspiration: Insects through art, science, and culture”. There were 2 unbiased speakers who introduced the debate topics as well as 4 teams who debated the following 2 topics: (i) Is forensic entomology viable in criminal case investigations and court cases today? and (ii) Are insects being treated ethically in scientific research? The teams prepared for about 8 months, debated their arguments, and shared their thoughts with the audience. The teams were judged by a panel and the winners were recognized at the ESA Student Awards Session during the annual meeting.

The student debates competition is an annual cross-examination competition organized and hosted by the Entomological Society of America (ESA) Student Affairs Committee (SAC) Student Debates Subcommittee (SDS) at the ESA annual meeting. This is an exciting opportunity for students to work together, hone their public speaking skills, and engage in an in-depth critical thinking process in front of a live audience. While the student debates may take place at the ESA annual meeting, the SDS starts communicating with the teams and the competing team members prepare their arguments well in advance, spending almost 8 months before the competition composing their arguments. 

The overall theme of the student debates and debate topics are determined by the SAC SDS and typically relate to the theme of the ESA annual meeting. Each topic within the student debates is introduced by an unbiased introductory speaker from the SAC SDS. Following this, 2 teams with different stances defend their position on the topic and then cross-examine the opposing team. The SAC SDS recruits ESA members as judges, and they score the teams based on the quality of their introduction, cross-examination, rebuttals, and responses to questions. The SAC and judges also consider the use of time, the strength of the supporting literature, abstract, and ability to meet deadlines leading up to the debates for the final scores.

The SAC emphasized on the ESA, ESC, and ESBC 2022 meeting theme—Entomology as inspiration: Insects through art, science, and culture—and identified 2 important topics highlighting popular entomological interests beyond research and education: (i) Is forensic entomology viable in criminal case investigations and court cases today? and (ii) Are insects being treated ethically in scientific research? In this article, we outline the 2022 student debates by first including the unbiased introduction for each topic followed by the teams’ responses on respective topics.

## Is Forensic Entomology Viable in Criminal Case Investigations and Court Cases Today?

### Unbiased Introduction by Sarah Elzay

Historically, forensic entomology, the use of insects (and, at times, mites and ticks, among other species) as evidence in legal proceedings, has important applications within 3 categories of forensic investigations: urban, stored-product, and medico-criminal (Lord and Stevenson 1986). The medico-criminal applications are those used in criminal investigations. One of the most important applications of forensic entomology is the estimation of minimum time since death, or minimum postmortem interval (PMI; Catts and Goff 1992, Amendt et al. 2004). Insects visit corpses to feed, live, or breed in, and different species will visit corpses in various states of decay (Abbott 1937, Mearns 1939). The distribution of insects across the globe enables forensic entomology techniques to be employed worldwide. As technology has advanced, identifying individual insect species as it relates to minimum PMI has improved the inferential nature of forensic entomology (Mona et al. 2019). Research into the effects of different climatic conditions has allowed forensic entomology to be employed worldwide. When insects are present on corpses, the use of forensic entomology is invaluable in minimum PMI estimations (Lutz et al. 2021a, Sardar et al. 2021). However, forensic entomology is imperfect and when used in the context of wrongful deaths, may raise ethical issues (Moreau 2021).

Successional data is inherently variable yet the research behind the successional data comes from academic settings that may fail to sufficiently study the variability that occurs in wrongful death scenarios (Michaud et al. 2012, Moreau 2021). Warming temperatures and precipitation changes caused by climate change may also impact the use of insects in forensic entomology. Climate change alters insect distribution and potentially phenological changes to insect succession. For example, the blowfly *Chrysomya albiceps* (Wiedemann) has experienced a range shift northward in Europe and the use of this species for estimating the site of death may no longer be appropriate in some cases (Grassberger et al. 2003). Additionally, guidelines and standards within the field of forensic entomology are nascent, leading to disorganized practice of forensic entomology (Amendt et al. 2007). Modern molecular techniques can be applied to the field of forensic entomology to improve the standardization of the field and accuracy of its application (Sardar et al. 2021, Singh et al. 2022).

Advanced molecular techniques including immunohistochemistry, spectroscopy, and flow cytometry may eclipse forensic entomology. However, continued improvement of entomological data, particularly in the context of variable climate conditions and climate change can improve forensic entomology and its application to criminal investigations. This debate addresses the future application of forensic entomology in forensic investigation.

## Team 1 Stance: Yes, Forensic entomology is viable in criminal case investigations and court cases today.

Team members: Kayleigh C. Hauri, John J. Ternest, Natalie Constancio, Scott Gula, Olivia M. Gearner

Faculty advisor: Dr. Zsofia Szendrei, Michigan State University

In court cases, a thorough investigation should examine all possible evidence to resolve the conflict between the opposing parties. This evidence is then evaluated by experts, lawyers, and opposing counsel; ultimately it is deliberated by a judge and jury. One type of viable evidence is forensic evidence, including forensic entomology. Forensic entomology is most widely recognized for evaluating the minimum time since death (minimum PMI) via insect colonization. However, it can also be applied to a wide range of cases including abuse and neglect (Benecke et al. 2004), poaching (Anderson 1999), trafficking of illegal substances (Crosby et al. 1986, Macedo et al. 2013), and establishing a suspect’s location (Parker 2007). This information provides crucial physical evidence which must be handled according to appropriate protocols, similar to DNA or fingerprints. Additionally, technological advances have made the applications of forensic entomology more wide-ranging and accurate than ever before. Therefore, forensic entomology is viable and crucial in criminal case investigations and court cases today.

Currently, a large body of research exists regarding insect development over a wide range of scenarios. These include temperature variation (Matuszewski and Mądra-Bielewicz 2016, Zhang et al. 2019), presence of drugs (Goff et al. 1989), and different environmental conditions (Tarone and Foran 2006, Wilson et al. 2014). If a body has been moved, DNA sequencing from the gut contents of maggots left behind at the crime scene can reveal the identity of the body (Wells and Stevens 2008). Additionally, gut contents of pubic lice transmitted during a sexual assault can reveal the identity of the assailant (Wells and Stevens 2008). Forensic entomology has also been used to prosecute poachers (Anderson 1999), identify the source of illegally imported goods (Crosby et al. 1986, Macedo et al. 2013), and establish the location of suspects (Parker 2007). Forensic entomology is regularly used in abuse and neglect cases of humans (Benecke and Lessig 2001, Benecke et al. 2004) and animals (Anderson 2013), especially to demonstrate whether maggot feeding occurred before death, pointing to neglect.

Although traditional forensic entomology largely involves insect species and life stage identification, technological advances in the field have exploded in recent years. One major area of advancement is the use of chemical ecology in applications of forensic entomology. Rapid evaporative ionization mass spectrometry (REIMS) in combination with model building can be used to distinguish morphologically similar insect species with extremely high accuracy (Wagner et al. 2020). Solid-phase microextraction (SPME) combined with gas chromatography–mass spectrometry (GC–MS) has been used to determine the species of blowfly eggs based on volatile profiles (Giffen-Lemieux et al. 2020). DNA methods have also seen significant advancements in recent years (Wells and Stevens 2008, Chimeno et al. 2019). For example, advances in DNA barcoding and sequencing methods have produced accurate, deployable techniques for determining the age of adult blowflies (Estévez et al. 2020). These advances will not only widen the range of data that can be analyzed, but they have also added tools that allow the identification of forensically important species which are morphologically similar.

The techniques and practices of forensic entomology are met with the scrutiny of the scientific peer review process that provides crucial advancement and refinement. In the context of court, forensic entomology retains the judicial standards of evidence evaluation present in all cases. This two-pronged process means that forensic entomology is not only viable but a crucial component in the fair and thorough evaluation of cases that include entomological evidence.

## Team 2 Stance: No, Forensic Entomology Is Not Viable in Criminal Case Investigations and Court Cases Today

Team members: Magdeline Anderson, Molly Edeburn, Brandon Hall, Jacqueline Maille, Mollie Toth

Faculty advisor: Dr. Kristopher Silver, Kansas State University

Forensic entomology can provide information that may lead to correct conclusions or accurate criminal investigation rulings. However, this relies on a highly trained forensic entomologist to arrive at crime scenes for observation, evidence collection, and subsequently provide expert reports or testimonies following established global guidelines that are informed by supporting studies about insect biology, insect ecology, and the application of techniques in specific situations (Barnes et al. 2022). Unfortunately, one or more of these requirements often remains unfulfilled, which leads to inaccurate evidential interpretation and conclusions resulting in subsequent improper conviction or acquittal of suspects. These outcomes demonstrate the lack of viability forensic entomology has in criminal investigations and court cases today.

Forensic entomology is as reliable as its regulations and training standards. Though European (Amendt et al. 2010) and American (Magni et al. 2013) professional forensic entomology societies provide published guidelines, there are no required regulations of facilities, certification, or verification of investigators. Inadequate and unregulated guidelines allow for untrained professionals to take entomological samples and evidence, which is common due to shortages of qualified professionals (Lutz et al. 2021b) and opens dispute in legal systems because evidence often is deemed sound from third-party review or court approval (Hall 2021). Misinterpretations made by nonprofessionals occur because of arthropod interference (Benecke and Lessig 2001, Sukontason et al. 2007) antemortem appearing like a weapon, cigarette scaring, chemical burns, or compromising blood spatter patterns (Viero et al. 2019). Also, uncommon crime scene species are often overlooked or dismissed in cases of abuse, neglect, and homicide involving wounds, bite marks, strangulation, and other torture methods (Benecke and Lessig 2001, Benecke et al. 2004, Sukontason et al. 2007, Amendt et al. 2011, Viero et al. 2019).

Although forensic entomology evidence should be handled and collected within the initial hours and days of criminal investigation at the crime scene to minimize inaccurate interpretations (Benecke et al. 2004, Cordner and Woodford 2020), evidence is often compromised when it is shipped to a professional or taken during autopsy (Lutz et al. 2021a). Further problems arise when contextual information is not urgently related to professionals. A 2015 case in China incorrectly estimated PMI based on 3-day-old maggots, even though they were collected from a fresh corpse (Wang et al. 2019).

Unfortunately, climate change continues to exacerbate forensic entomology issues by altering arthropod behavior (Nawoichik and Johnson 2016), PMI, and ecological shifts in populations (Vanin et al. 2008). But criminal case investigations usually disregard these environmental conditions that have a tremendous impact on insect behavior and development (Nihal 2016). To improve regional accuracy, routine species surveys with a variety of climate components are needed to yield reference data for applicable use (Vanin et al. 2008). Such forensic entomology research requires labor-intensive dynamic studies that include a multitude of crime scene conditions. Yet, the lack of modern geographic depictions (Barnes et al. 2022), spatial scales (Boudreau and Moreau 2022), appropriate replications (Hall 2021), statistical applications (Amendt et al. 2011, Moreau 2021), and inferential studies make forensic entomology less applicable (Michaud et al. 2014). Advancements in molecular techniques can make insect identification more accurate, but some key species cannot be distinguished with routine molecular barcoding techniques (Wells et al. 2007, Whitworth et al. 2007, Tourle et al. 2009).

The magnitude of these discrepancies creates enough valid hesitancy for there to be widespread concern surrounding FE, particularly since these techniques are used in making decisions that could be an injustice including incarceration or death. With many outside impacts including differing national judicial systems, differing species, and climate change, transformations are necessary for feasible forensic entomology practices. Stronger global communication and framework are needed for forensic entomology to be an applicable science that is fluid and flexible to modern innovations in the future. To allow for easy integration of future innovations and adaptation in the limits of policy and practices for reliable governance in criminal trials.

## Are Insects Being Treated Ethically in Scientific Research?

### Unbiased Introduction by Elizabeth Bello

Humans interact with insects in a variety of contexts: in art and entertainment, as food and feed, as pests or biological control agents, and for educational, medical, or research purposes. The use of nonhuman animals for human interests can be ethically fraught when those animals have their own interests—namely, when they are sentient, or capable of suffering (Singer 2002). It is commonly accepted that the welfare of sentient organisms deserves some moral consideration, though different ethical theories have different demands. Animal welfare theories suggest that the interests of animals can be traded for other goals, such as the pursuit of knowledge or to reduce human suffering, if the animal research subjects are treated humanely (i.e., with care and consideration for their well-being) (Baracchi and Baciadonna 2020). Meanwhile, animal rights theories argue that some basic interests should never be violated for such goals (Regan 1986).

In support of the animal welfare view, numerous legislations have been passed to protect the welfare of vertebrate research subjects. These protections do not, however, extend to insects. This is due to the traditional assumption that insects are not sentient, and thus cannot feel pain, justifying their exclusion from moral consideration. However, recent research has suggested that insects meet many of the key criteria considered relevant to the capacity for sentience. In addition, some of the assumptions underlying publications arguing against the capacity for pain in insects have been proven incorrect (e.g., arguments in Eisemann et al. 1984 and Adamo 2019, refuted by Tracey et al. 2003 and Li et al. 2020). Given that estimates suggest well over 100 million insects are used annually in research, ethical consideration of their treatment is urgent (Rowe 2020a).

Several researchers have argued that insects lack the neuroanatomical complexity required to support sentience (Adamo 2019, Key et al. 2021). If insects are not sentient and therefore incapable of experiencing feelings or sensations like pain and discomfort, then their treatment cannot be morally objectionable. In addition, using insects often allows researchers to avoid experimentation on organisms that are sentient, such as mice (Sandall and Fischer 2019). In this case, using insects reduces net harm to animals. Other researchers argue that insects can be used ethically in research even if they are sentient. For example, the use of sentient animals could be considered ethical if the benefits to humans either (i) outweigh the harm caused to insects during research, or (ii) if harms are minimized to only those necessary to obtain the research goal. Additionally, some posit that the conditions used to rear insects in research already support reasonable welfare, as promoting high survival is typically desired while conducting research (similar arguments are made for mass-rearing; Van Huis 2021).

Others have argued that recent research suggests insects may be, or are likely to be, sentient; in this case, moral caution is warranted to avoid potentially causing catastrophic harm to many beings (Birch 2017, Lambert et al. 2019, Mikhalevich and Powell 2020). Several scientists have argued for a version of the precautionary principle, treating insects with respect and care to protect their potential welfare (Lockwood 1987, Sandall and Fischer 2019, Trietsch and Deans 2018). Additionally, while using insects may allow researchers to avoid using vertebrates, we must consider all the organisms’ welfare as a trade-off. In this case, replacing (fewer) vertebrates with (more) insects may result in a net welfare harm to animals overall. Currently, insects are not subjected to welfare regulations, in research or elsewhere. This debate will further address the ethical treatment of insects in scientific research.

## Team 1 Stance: Yes, Insects Are Being Treated Ethically in Scientific Research

Team Members: Arjun Khadka, Ethan Doherty, Tyler Musgrove, Tiago Silva, Alexia Desoto

Faculty Advisor: Blake Wilson, Louisiana State University

Treatment of insects in research is considered ethical if the expected benefits of the research outweigh harm to research subjects (Grimm et al. 2019). Entomological research is imperative because of the propensity of insects to impact the world. Arthropod pests destroy an estimated 18–20% of global annual crop production at an estimated economic loss of >$470 billion USD (Sharma et al. 2017). Structural insect pests including termites cause an annual economic impact of $2–3 billion from property damage in the US alone (Govorushko 2019). Arthropod vectors of human disease cause 7 million deaths annually (WHO 2020). Given the scope of their influence, the use of insects as research subjects is not only justified, but critical to maintaining human subsistence and ecological balance (Adamo et al. 2012).

Advancements in food security and reductions in health risks are the direct results of entomological research. The eradications of the boll weevil, *Anthonomus grandis*, and the screwworm, *Cochliomyia hominivorax*, are 2 examples of the benefits of insect research (Vargas-Teran et al. 2005, Raszick 2021). The use of insects in research is also common among other disciplines such as genetics, engineering, and ecology (Price 2003, Adamo et al. 2012, Govorushko 2019, Rothschild 2020). Studies of *Drosophila* have revealed that 75% of genes responsible for human disease are conserved across animals (Adams et al. 2000). Studies of termite mounds led researchers to develop effective ventilation systems that can be implemented in developing countries to mitigate energy costs (Govorushko 2019). Ecological insect studies have been foundational to biological control and our understanding of predator–prey relationships (Price 2003).

Despite the widespread use of insects in science, the impact to research specimens is miniscule compared to their treatment in urban or agricultural environments. There are ten quintillion (10^18^) insects alive on earth at any given time (Smithsonian 1996). Of these, 0.01–10 quadrillion are killed by insecticides (Rowe 2020b) and 32.5 trillion are killed by cars annually (Messenger 2018), all in the absence of ethical regulations. These numbers are orders of magnitude more than the estimated 100 million to 10 billion used in research annually (Rowe 2020b).

Additionally, insects offer a more ethical alternative to the use of vertebrate animals as test subjects. The most widely accepted guidelines and studies of animal ethics advocate for the use of invertebrate research subjects whenever possible (Festing and Wilkinson 2007, Fenwick and Gauthier 2009). There is strong evidence that vertebrates may experience pain and consciousness in a similar way to humans, however, the evidence to suggest that insects experience these same phenomena is considerably weaker (Adamo 2016). Their ability to respond to damaging stimuli could be categorized as nociception. While the degree to which insects are sentient is unclear, they are unlikely to rival sentience of any vertebrates (Fischer 2016). Insects can then be considered less sentient and have a reduced perception of pain relative to vertebrates. For these reasons, invertebrates were exempt from the Office Laboratory Animal Welfares regulations developed by national experts in research ethics and are considered appropriate research alternatives to vertebrates (OLAW 2021).

Finally, regulating the use of insects in research would constrain the scientific process. The requirements of IACUC and other regulatory bodies would pile additional burdens onto the researcher’s time, money, and effort (Harvey-Clark 2011, Pritt et al. 2016). Moreover, these regulations would be couched in the personal views of those serving on ethics review committees, constraining entomologists to the politics of a few (Ideland 2009). Any constraints on entomology may negatively impact human welfare. Therefore, we consider the current practices and uses of insects in research to be ethical, as the advancements gained outweigh detriments.

## Team 2 Stance: No, Insects are Not Being Treated Ethically in Scientific Research

Team members: Emily Rampone, Dowen Jocson, Mario Luppino, Kellen Pautzke, Camille Wagstaff

Faculty advisor: Jeb Owen, Washington State University

The use of animals in research has been essential to advancements in our knowledge about the natural world. The standards of treatment for those animals have shifted dramatically with increased understanding of animal pain perception and changes in public opinion over the centuries. In the 17th century a concept emerged that capacity for suffering has more value than the ability to reason, resulting in early regulations to improve animal welfare (Duncan 2019). Over the past 60 years animal welfare regulations have expanded with the development of Russell and Burch’s guidelines that now dominate the way researchers think about ethics in animal research (Festing and Wilkinson 2007, Garrett 2012).

Arguments against welfare regulation in invertebrate research have historic parallels in vertebrate research, such as concerns regarding regulatory burden (government interference in research) and the cost of research (Vasbinder and Locke 2016). Despite these concerns, vertebrate regulations have not reduced scientific output or hindered well-designed research (Drinkwater et al. 2019). In fact, regulation of animal welfare has improved research quality because it reduces variation and unpredictability among animal subjects, increasing statistical power, and experimental reproducibility (Tannenbaum and Bennett 2015, Mohan and Huneke 2019). Versions of Russell and Burch’s 3Rs (Replace, Reduce, Refine) are now followed internationally to guide animal use (Russell and Burch 1959a, 1959b, Tannenbaum and Bennett 2015). Many countries facilitate this by creating animal care and use committees, which function partially as ethics committees, reflecting scientific and public opinion, and partially to apply existing legislation to animal welfare in research (Harvey-Clark 2011, Hansen 2013). Invertebrate animal research remains mostly unexamined and unvetted prior to being conducted.

Even with increased focus on animal welfare in science, invertebrates like insects have been excluded from ethical consideration using based on antiquated arguments based on and limited understanding of sentience and cognition (Fischer and Larson 2019, Freelance 2019). However, recent studies demonstrate that some insects have the capacity to experience pain and distress (Gibson et al. 2015), as well as the ability for higher cognitive abilities like learning (Blackiston et al. 2008, Giurfa 2013). Additionally, the discovery of analogous brain structures between insects and vertebrates supports the potential for insect subjective experience, indicating a need for insect consideration in animal welfare guidelines (Freelance 2019). While we believe insects meet the criteria for sentience, the demonstration of sentience should not be a requirement for ethical consideration (Knutsson and Munthe 2017, Villamor Iglesias 2021).

Pioneering insect researchers have highlighted a need to consider potential harmful research practices and reduce suffering despite a lack of legislation or public acknowledgment (Wigglesworth 1980, Lockwood 1987). We propose that given our increased understanding of insect biology and behavior, it is necessary and possible to adapt the 3Rs to insect research. Accomplishing this will involve critically examining invertebrate research practices: reducing specimens collected and used, replacing insects with computer modeling when possible, and refining and standardizing global research methods. To do this, we need to maximize the information gained from the number of specimens utilized, while reducing the overall distress they feel (Tannenbaum and Bennett 2015).

Investing in standardized research methods will improve scientific research and public opinion on scientific research (Drinkwater et al. 2019). Standardizing insect research protocols would ensure research reproducibility, reflect advancements in humane insect treatment, and provide equitable consideration among all animals used in research (Horvath et al. 2013). While much of ethics has changed over the last 300 years, the notion that our treatment of other species is a reflection of our own humanity has been a throughline. It is time to eliminate outdated notions of invertebrate inferiority and recognize insects for ethical consideration in research for ourselves and the subjects we study.
